# Development of a malignant hyperthermia protocol

**DOI:** 10.1186/1753-6561-9-S1-A32

**Published:** 2015-01-14

**Authors:** EYH Yeung, J Munroe

**Affiliations:** 1 Faculty of Pharmaceutical Sciences, The University of British Columbia, Vancouver, British Columbia, Canada; 2 Support Services Facility, Fraser Health Authority, Langley, British Columbia, Canada

## Background

Malignant Hyperthermia is a life-threatening condition triggered by exposure to certain general anaesthetics (halothane, sevoflurane, and desflurane) and depolarising muscle relaxants (suxamethonium). Malignant hyperthermia happens primarily due to mutation of the ryanodine receptor type 1 (RYR1), located on the sarcoplasmic reticulum in myocytes. This mutation leads to increase in calcium release, muscle contraction, and heat production. Dantrolene, a skeletal muscle relaxant, is the drug of choice for malignant hyperthermia because it binds to RYR1 and thereby reduces the calcium released from the sarcoplasmic reticulum.[1] Dantrolene has been shown to significantly reduce mortality when given promptly.[2,3] A hospital is recommended to keep a minimum stock of 36 dantrolene vials, which provides 720 mg of dantrolene sufficient for a 70-kg person.[4] This study investigates whether the hospitals in the region of Fraser Health Authority, Canada, have sufficient dantrolene vials in stock.

## Methods

A visit was made to the eleven hospitals’ operating rooms in the region. The expiry date and location of the dantrolene vials were recorded. The operating room staff were interviewed to determine their knowledge on the treatment procedure of malignant hyperthermia.

## Results

Four of the hospitals were found to have less than 36 vials of dantrolene in the operating rooms. Most of the staff never treated patients with malignant hyperthermia and did not know the reconstitution procedure of dantrolene.

## Conclusions

A dantrolene cart, which consisted of 36 vials of dantrolene and a simplified reconstitution instruction, was determined to be necessary. The cart would also have other supplies for management of malignant hyperthermia, including furosemide, lidocaine, calcium chloride, dextrose, sodium bicarbonate, sterile water, regular human insulin, and syringes. Routine stock quantity and expiry date checks would be carried out. One of the hospitals was recommended to stock 72 vials of dantrolene. The extra vials would be transferred to another hospital during shortage of dantrolene in emergency situations. Quarterly drills on reconstitution of dantrolene and treatment of malignant hyperthermia were also deemed to be necessary.
